# Accelerated mass extinction in an isolated biota during Late Devonian climate changes

**DOI:** 10.1038/s41598-021-03510-6

**Published:** 2021-12-21

**Authors:** Jaleigh Q. Pier, Sarah K. Brisson, J. Andrew Beard, Michael T. Hren, Andrew M. Bush

**Affiliations:** 1 Department of Ecology and Evolutionary Biology, University of Connecticut, 75 North Eagleville Road, Storrs, CT 06269-3043 USA; 2 Earth and Atmospheric Sciences Department, Cornell University, 4 College Ave, Ithaca, NY 14850 USA; 3 Department of Geosciences, University of Connecticut, 354 Mansfield Road, Storrs, CT 06269-1045 USA

**Keywords:** Ecology, Evolution, Ecology

## Abstract

The fossil record can illuminate factors that contribute to extinction risk during times of global environmental disturbance; for example, inferred thermal tolerance was an important predictor of extinction during several mass extinctions that corresponded with climate change. Additionally, members of geographically isolated biotas may face higher risk because they have less opportunity to migrate to suitable climate refugia during environmental disturbances. Here, we investigate how different types of risk intersect in the well-preserved brachiopod fauna of the Appalachian Foreland Basin during the two pulses of the Frasnian–Famennian mass extinction (Late Devonian, ~ 372 Ma). The selectivity of extinction is consistent with climate change (cooling) as a primary kill mechanism in this fauna. Overall, the extinction was mild relative to other regions, despite the many endemic species. However, vulnerable taxa went extinct more rapidly, during the first extinction pulse, such that the second pulse was insignificant. These results suggest that vulnerable taxa in geographically isolated biotas face heightened extinction risk at the initiation of environmental stress, but that taxa in other regions may eventually see elevated extinction risk if environmental stress repeats or intensifies.

## Introduction

Survival through major climatic and environmental perturbations may depend on a species’ ability to migrate to habitable refugia^5^, but many species face climatic, environmental, and geographic barriers to migration. Inability to migrate may be particularly acute in the modern world, as climate change coincides with land-use changes and habitat fragmentation. The fossil record is increasingly used to evaluate potential extinction risk factors during mass extinctions, and it has the potential to illuminate how different types of risk interact^1,2,6^. To this end, we document the fate of a semi-isolated, regional brachiopod fauna during the Frasnian–Famennian mass extinction in the Late Devonian. Two pulses of extinction were separated by about 800,000 years^3,4^ (the Kellwasser Events), with the second pulse viewed as more severe^3,4^. In general, both pulses are associated with global cooling, the expansion of low-oxygen conditions in marine environments, and carbon cycle disruption^7–13^, although some studies indicate that they were not identical (e.g., cooling may have been more severe during the first pulse, and the second cooling event may have been preceded by warming)^14,15^.

These pulses of extinction drove a decline in global standing diversity in the oceans and considerable ecological change^16^, and lowered speciation rates may have exacerbated the decline in diversity in North American brachiopods^17^. Despite a general increase in cosmopolitanism at this time^17^, extensive new fossil collecting and revised stratigraphic correlations indicate that the pre-extinction brachiopod fauna in the Appalachian Foreland Basin^18,19^ consisted mostly of endemic species (70%), in accordance with previous observations that this benthic fauna was distinct from similar-aged assemblages from central and western North America^20^. The Appalachian Basin was partially isolated from other North American marine basins by land barriers and by differences in habitat: the Appalachian seaway received high siliciclastic sediment input from the nearby Acadian Mountains, and substrate is a major control on brachiopod distributions. In addition, fauna in epicontinental seas may have faced added climatic barriers to migration^21^.

**Figure 1:**
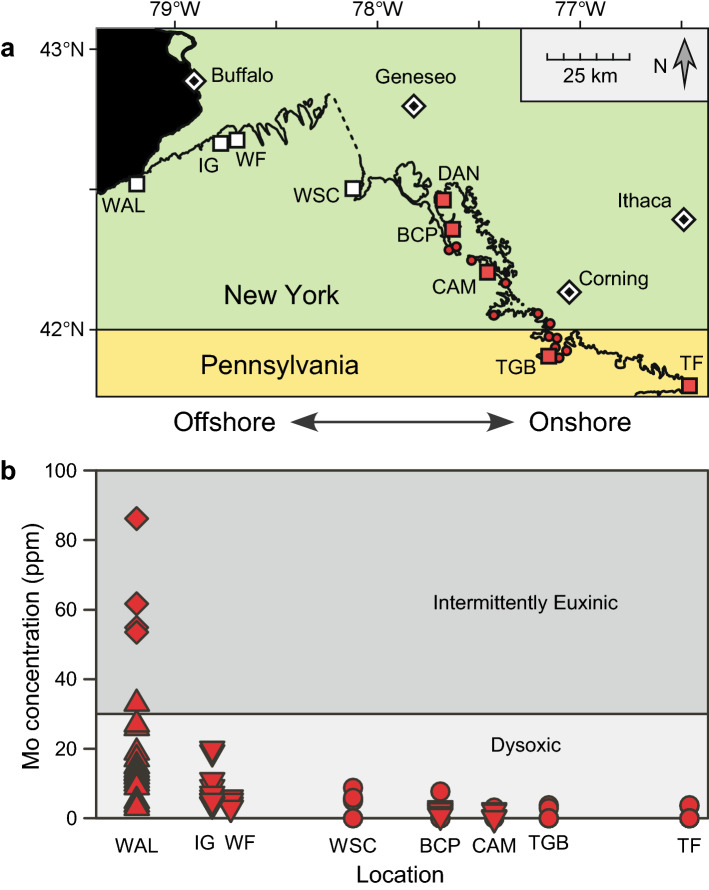
Locations and paleoenvironmental proxy data. (**a**). Map of study area. Large red squares represent measured stratigraphic sections from which numerous samples were collected, and small red circles represent additional sampling localities (see Table S1 for coordinates). White squares represent sections sampled only for geochemistry due to rarity of macrofossils. (**b**). Mo concentrations from the Pipe Creek Formation as a proxy for oxygen level during the first extinction pulse. In the onshore localities examined here (WSC through TF), Mo concentrations were below the detection limit in some samples. The “Dysoxic” and “Intermittently Euxinic” fields follow Ref. ^60^ and ^61^. Sources of data: circles = this study; diamonds = Ref. ^38^; upward pointing triangles = Ref. ^8^; downward pointing triangles = Ref.^33^. See Supplementary Table S2 for data. Figure 1a plotted in R with package rgdal and modified in Canvas 11. Map data was downloaded from http://www.nysm.nysed.gov/research-collections/geology/gis and also ^62,63^.

To examine the timing and selectivity of the extinction, we intensively collected rhynchonelliform brachiopods from numerous stratigraphic sections along a shallow-to-deep habitat transect in Pennsylvania and New York (Fig. 1a, Supplementary Table S1). Siliciclastic deposition dominated throughout the extinction interval. Brachiopods were abundant in the Devonian, and their calcitic shells preserved readily. The primary dataset consists of 7,933 specimens from 94 bulk samples from the Wiscoy Formation (prior to the first extinction pulse) and 7,095 specimens from 97 bulk samples from the Canaseraga Formation (between the two pulses and immediately after the second pulse). We also collected many thousands of additional fossils that were not counted but were used to verify stratigraphic ranges and identify victims and survivors.

Given the temporal coincidence of global cooling and the demise of tropical taxa, climate change has been suggested frequently as a proximal kill mechanism^22–24^. Ocean anoxia is also frequently invoked, in addition to several other potential factors^10,25–30^. To evaluate the potential importance of cooling and anoxia as kill mechanisms in this basin, we evaluated extinction selectivity of brachiopods using exact logistic regression^31^. Sensitivity to climate change is often assessed in fossil species using paleolatitudinal range; although it does not indicate absolute temperature tolerance, it has been useful as a proxy for relative adaptation to warmer versus cooler temperatures^1,5^ . However, applying this approach to Frasnian marine taxa is complicated by a dearth of high-latitude faunas, making it more difficult to assess adaptation to cooler waters. As a result, we used two different approaches to gauge relative temperature sensitivity; although neither is a perfect proxy for temperature tolerance, they are largely independent and are unlikely to suffer the same set of biases. The species-level indicator was based on the paleolatitudinal distribution of individual species, and the order-level indicator was based on the paleolatitudinal distribution of brachiopod orders in the Paleobiology Database (paleobiodb.org). Although species within orders do, of course, vary in their paleolatitudinal ranges, some orders are uncommon at high latitudes in the Devonian, suggesting that members of these orders might have had limited ability to cope with colder temperatures^22,24^ (see Methods for further discussion).

Prior studies indicate that anoxia in this basin is associated with deeper-water, offshore habitats^8,9,32^; the movement of these environments upslope during transgressions is recorded by regional deposition of black/dark gray shale units, like the Pipe Creek Formation, which marks the first extinction pulse. Geochemical data indicate that the first extinction pulse was associated with dysoxic conditions and occasional intervals of anoxia, with evidence of anoxia increasing towards the offshore; euxinia was only present in the deepest parts of the basin. Anoxia was more prominent during the second extinction pulse^33^.

If anoxia spreading from the offshore caused extinctions in the Appalachian Basin fauna, they are likely to be concentrated in more offshore settings; species living in shallow water are less likely to be exposed to low-oxygen conditions, as oxygen more readily mixes into shallower water. We thus used onshore-offshore habitat preference as a predictor of exposure to anoxia. Habitat preference in the pre-extinction fauna was measured using non-metric multidimensional scaling (NMDS)^34–36^ of the 94 bulk fossil samples from the Wiscoy Formation, which immediately precedes the first extinction pulse, taken along a transect that encompassed all habitats in which brachiopods were common (Fig. 1a). We also evaluated paleo-oxygenation levels in the Pipe Creek Formation using trace metal concentrations, extending previous studies^33^ eastward into even more proximal settings. Body size and abundance (average proportion within samples) were also tested as predictors.

## Results

**Figure 2:**
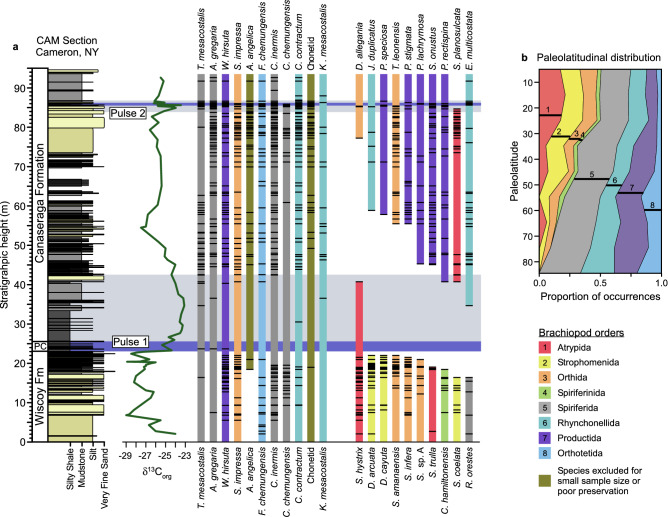
Stratigraphic and paleolatitudinal distributions of brachiopods. (**a**). Stratigraphic section through the two extinction pulses (“Pulse 1” and “Pulse 2”) at location CAM showing δ^13^C_org_ excursions and brachiopod species occurrences (horizontal black dashes). Not all species are present at this section. Colored vertical bars mark the total known stratigraphic ranges of species and are extended to the base or top of the section for species known to occur lower or higher based on data from other localities. Color indicates taxonomic order for each species. Full species names are given in Supplementary Table S4. (**b**). Paleolatitudinal distribution for brachiopod orders during the Devonian, showing only orders included in the regression analysis (data for northern and southern hemispheres combined). Black lines indicate the weighted averages of the paleolatitudinal distributions of the orders.

The first pulse of extinction was substantial, with 55% of species extirpated (Fig. 2a), but the second pulse was negligible, with only one member of the original Wiscoy fauna dying out (*Cyrtospirifer chemungensis*). Following the first pulse of extinction, there was an influx of new species, and one of these immigrants also died out in the second pulse (*Spinatrypa planosulcata*, Fig. 2a). The last occurrences of the victims of the first pulse were spread out over the upper several meters of the Wiscoy Formation (Fig. 2a), which we attribute to the Signor–Lipps effect and facies effects^37^ (for comparison, the highest Wiscoy occurrences of survivor species were also spread out over several meters).

**Figure 3:**
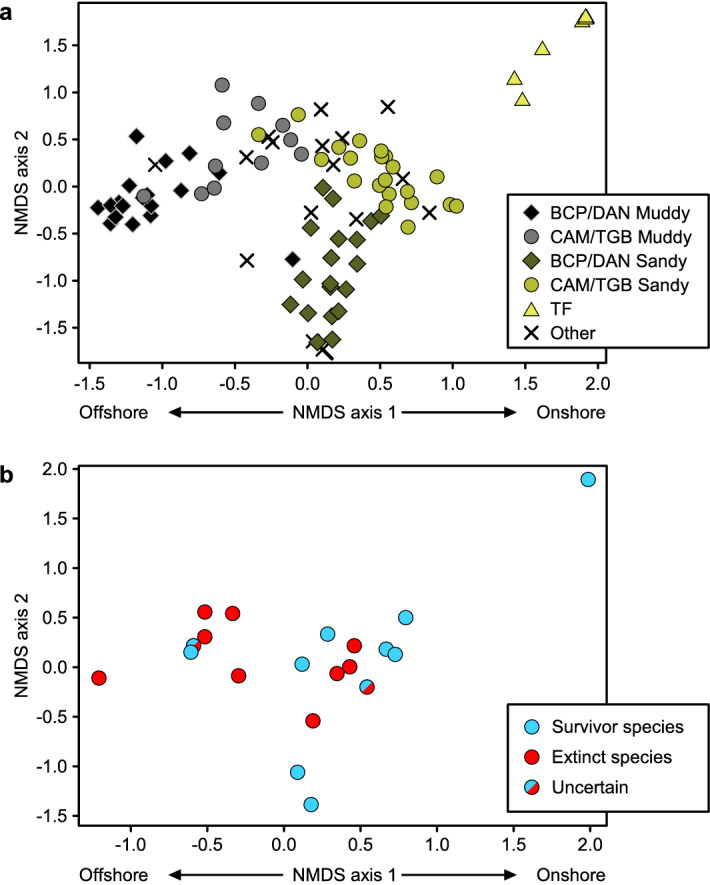
NMDS ordination of the brachiopod fauna from the Wiscoy Formation, which immediately precedes the first extinction pulse. (**a**). Samples labeled by locality and informal stratigraphic unit (offshore “Muddy Member” or onshore “Sandy Member”). Samples that plot at the negative end of NMDS Axis 2 contain a high abundance of *Ambocoelia gregaria*, which has been described as an opportunistic species that does not fall cleanly along onshore-offshore gradients in ordinations^34^. (**b**). Species labeled as victims or survivors of the first extinction pulse.

At one or more localities, *Spinatrypa hystrix* and *Schizophoria amanaensis* last occurred above the Pipe Creek Formation (the dark shale unit associated with the first extinction pulse) but within the δ^13^C_org_ excursion that marks the Earth-system disturbance associated with the extinction (e.g., *S. hystrix* in Fig. 2a). These species could be considered survivors that perished soon after the first pulse of extinction for unrelated reasons (e.g., if the extinction kill mechanism only operated during deposition of the Pipe Creek), or they could be considered victims (e.g., if the kill mechanism operated throughout the biogeochemical disturbance); this complexity was revealed by sampling multiple stratigraphic sections along a paleodepth transect^37^. Therefore, we ran the regression analyses twice, counting these species both as victims and as survivors, with the same result (Table 1). Given that few species went extinct in the second extinction pulse, we only analyzed extinction selectivity during the first pulse. Position on the first NMDS axis was strongly related to onshore-offshore position, as indicated by (1) samples from more offshore paleoenvironments (the informal “Muddy Member”) plotting to the left of samples from more onshore paleoenvironments (the informal “Sandy Member”), and (2) samples from more western (distal) localities plotting to the left of samples from more eastern locations within the same member (Fig. 3a). Therefore, a species’ score on NMDS axis 1 was used as a proxy for relative onshore-offshore habitat preference, with more negative scores indicating a more offshore habitat. Position on NMDS axis 2 was related to the abundance of *Ambocoelia gregaria*, which occurs in great abundance in certain environments and has been described as an opportunistic species^34^ (Fig. 3).

**Table 1:** Exact logistic regression results of variables predicting the likelihood of extinction of brachiopod species (see Methods).

a. Parameter	Paleolatitude Index (Composite Metric)	Body Size	Onshore-offshore Habitat Preference	% Abundance
Odds Ratio	0.11	0.99	4.13	0.41
*p* value	0.0002	0.977	0.237	0.601
Confidence Interval	(− 7.28, − 0.78)	(− 0.51, 0.23)	(− 0.94, 6.77)	(− 6.10, 4.87)

b. Parameter	Paleolatitude Index (Composite Metric)	Body Size	Onshore-offshore Habitat Preference	% Abundance
Odds Ratio	0.29	0.97	2.32	0.26
*p* value	0.009	0.680	0.397	0.1653
Confidence Interval	(− 2.99, − 0.26)	(− 0.21, 0.12)	(− 1.22, 3.02)	(− 3.87, 0.53)

(**a**) Analyses run with *Spinatrypa hystrix* and *Schizophoria amanaensis* as victims. Odds ratio = 16.18,* p* value = 0.003. (**b**) Analyses run with *Spinatrypa hystrix* and *Schizophoria amanaensis* as survivors. Odds ratio = 9.78, *p* value = 0.044. The Paleolatitude Index was the only significant predictor (i.e., confidence interval does not include zero).

Of the species included in the analysis, 30% also occurred in basins reconstructed at lower latitudes, thus meeting our species-level criterion for low-latitude affiliation (Supplementary Table S3). For the order-level analysis of paleolatitudinal distribution based on the Paleobiology Database, the atrypids had the strongest affiliation for low latitudes, followed by the strophomenids, orthids, and spiriferinids (Fig. 2b; Supplementary Table S3). These orders were uncommon at high latitudes relative to the productids, rhynchonellids, orthotetids, and spiriferids. In univariate tests, both the species-level and order-level indicators of low-latitude affiliation were significantly and positively related to extinction in the first extinction pulse (Supplementary Fig. S1; Supplementary Table S5). In fact, all species that extended into lower-latitude basins perished, as did most species belonging to the orders that were most common at lower latitudes (Atrypida, Strophomenida, Spiriferinida and, to a lesser extent, Orthida; Fig. 2). No other variable was significant in univariate tests, including onshore-offshore habitat preference, mean body size, and mean local relative abundance (Supplementary Fig. S1; Supplementary Table S5).

For the exact logistic regression, we combined our two indicators of low-latitude affiliation into a composite metric, although using just one or the other produced the same results. The regression indicated, again, that extinction was only predicted by low-latitude affiliation (Table 1).

Geochemical analysis of our stratigraphic sections supports prior conclusions that anoxia during the first extinction pulse (Lower Kellwasser Event) did not penetrate shallow-water habitats^33^ (Fig. 1b). Anoxia, and perhaps euxinia, was intermittently present in the most offshore habitats in western New York where macrofossils are rare^8,38^. However, more onshore habitats were characterized by dysoxia, which decreased in intensity shoreward (Fig. 1b). Similarly, bioturbation and trace fossils are common at more onshore sections as far west as location BCP^18,33^, and body fossils are present at TGB, indicating dysoxia rather than anoxia^18^, similar to other dark shales in the region^39^. Enrichment factors^9^ for Mo, U, and V similarly showed generally low enrichment, decreasing shoreward (Supplementary Table S2).

## Discussion

During the Frasnian–Famennian transition in the Appalachian Basin, almost all brachiopod species losses occurred during the first pulse of extinction, such that it can scarcely be described as having two pulses. All members of the Atrypida and Strophomenida that were present in the Wiscoy Formation were eliminated in the first extinction pulse, with one atrypid migrating in afterward (Fig. 2a). This highly selective first pulse of extinction drove a notable change in the taxonomic composition of the fauna towards dominance by productids, spiriferids, and rhynchonellids (Fig. 2a).

In fact, patterns of extinction in the Appalachian Basin were quite unusual – for example, numerous strophomenids and atrypids survived the first extinction pulse in the central and western United States, despite a larger overall proportion of species dying out^20,40^. The major change in taxonomic composition of the brachiopod fauna did not occur elsewhere until the second extinction pulse, which was large and devastating (e.g., only 4 of 35 New Mexico species are known to survive the second pulse^41,42^). Diversity also declined during both extinction pulses in shallow marine basins in Europe, with the lowest diversity reached after the second pulse of extinction^43–45^. Across much of Europe and Southern China, major losses of atrypid and strophomenid brachiopods occurred during the second extinction pulse^46–48^.

As noted above, examining extinction selectivity with respect to paleolatitudinal range is complicated by a lack of high-latitude brachiopod faunas in the Frasnian (Supplementary Fig. S2); given this constraint, we used two independent metrics to characterize sensitivity to climate change. Although both metrics have potential drawbacks (see Methods), it is notable that both gave a strong signal that low-latitude affiliation was preferentially associated with extinction, consistent with climate change as a proximal kill mechanism for this fauna. Additional quantitative studies of extinction selectivity could be extremely valuable in establishing further connections between additional traits and extinction risk, thus providing a fuller view of the links between environmental and biotic change at this time.

Given that few species went extinct in the second extinction pulse, we did not test for selectivity. However, cooling as a proximal kill mechanism is consistent with the fact that one of the only losses was *Spinatrypa planosulcata*, which migrated in after the first pulse of extinction and meets both criteria for low-latitude affiliation (Fig. 2b).

Although anoxic conditions were at least intermittently developed during the extinction interval in more distal settings^8,9,33^ in New York, both trace metal (Fig. 1b) and faunal data suggest that these conditions declined in importance in more proximal settings^18,33^. Likewise, brachiopod extinctions were not concentrated in any particular habitat zone, as might happen if anoxia were concentrated in certain habitats (Table 1; Fig. 2b). Critically, the Pipe Creek Formation corresponds with a transgression in which all facies would have shifted upslope, and there is no evidence that low oxygen conditions had an unusual paleoenvironmental distribution at this time. Presumably, brachiopod species were able to track their preferred habitats during these sea level changes^49^. These results do not rule out a role for anoxia in other regions, nor a secondary role in this region.

Numerous factors contribute to a species’ extinction risk^6^, with thermal tolerance important in many mass extinctions associated with climate change^1,2^. Despite the large number of endemic species, extinction risk was not higher in the Appalachian Basin than in other regions—in fact, overall extinction magnitudes were lower than in other parts of North America^20,40,42^. Rather, the species that were vulnerable to environmental perturbation were eliminated in the first pulse, with the surviving fauna persisting relatively untroubled through the second pulse. In other words, in this biogeographic setting, the “low-hanging fruit” were eliminated in the first pulse, such that the second pulse was almost non-existent. In contrast, in other parts of the world, many members of the most vulnerable clades (atrypids, strophomenids) survived the first pulse but succumbed to the second. It is worth noting that current global paleontological databases lack the temporal resolution to examine extinction patterns at this level of detail, highlighting the continued usefulness of detailed local and regional studies. It is also worth emphasizing that larger geographic ranges did not buffer against extirpation in this fauna^50^ – species whose ranges extended to other basins actually had higher extinction risk.

These results suggest that multiple sources of extinction risk should continue to be evaluated for modern organisms, including both species-level traits and biogeographic context. However, the interaction among these sources of risk may depend critically on the time scale of observation and of environmental disruption. In the endemic-rich, semi-isolated fauna studied here, extinctions were concentrated at the onset of environmental disturbance, whereas elsewhere, substantial extinction extended throughout both disturbances. If environmental disruption and climate change are temporally persistent and/or recurrent, peak extinction intensity may occur non-synchronously in different geographic settings as vulnerable species are progressively eliminated in different biogeographic settings. These results also suggest that, at the global level, extinction can be mitigated by limiting the persistence and recurrence of adverse conditions.

## Methods

The positions of the first and second extinction pulses were established by biostratigraphy^19^ and carbon isotope stratigraphy (Fig. 2a). The Wiscoy Formation immediately precedes the Pipe Creek Formation, which is temporally equivalent to the first extinction pulse (Fig. 2a). The second extinction pulse corresponds to a thinner shale bed in the upper Canaseraga Formation (Fig. 2a). A total of 94 bulk fossil samples were collected from the Wiscoy Formation from 17 localities representing measured stratigraphic sections as well as smaller outcrops (Fig. 1a, Supplementary Table S1)^19^. These samples contained 7,933 rhynchonelliform brachiopod fossils from 26 species, with at least 30 individuals per sample. All samples represent a single bed or a few adjacent beds of similar paleoenvironment. Identifications were based on several sources that have illustrated the brachiopod fauna^18,51–53^. To avoid double-counting of individuals, parts and counterparts of fragmented rock samples were compared, and only brachiopods with at least half a valve visible were counted.

Chonetid brachiopods were excluded because they were small and often not well preserved in coarser lithologies; in particular, molds of chonetids in sandstones were frequently difficult to identify to the species level. Prior works suggest that two species are present^53^. Species with very low total abundance across Wiscoy samples (< 10 specimens) were also excluded from analysis (See Supplementary Table S3 for species included in analyses and Supplementary Table S4 for species present across both pulses; also presented in Fig. 2a). Including these taxa would only strengthen the results, as the excluded taxa matched patterns seen in other taxa (i.e., low-latitude affiliation associated with extinction). We could only include species in the regression analyses that occurred in our Wiscoy Formation samples, and several species reported in the literature did not occur in our samples. Had we been able to include these species, it would likewise have strengthened the results, as they all survived the extinction and all had non-tropical affinities according to our metrics (e.g., *Praewaagenoconcha speciosa*, *P. lachrymosa*).

We divided the Wiscoy into two informal units that represent different paleoenvironments (Figs. 2a). At the DAN and BCP sections (Fig. 1a), the “Sandy Member” consists of hummocky- or swaley-bedded, very fine sandstones with interbedded mudstones, indicative of storm reworking and interpreted as deposited just above mean storm weather wave base. To the southeast at CAM and TGB (Fig. 1), the “Sandy Member” has less mudstone and more amalgamated, swaley-bedded sandstone; the paleoenvironment is interpreted as above storm-weather wave base but below fair-weather wave base^18^. Farther east (location TF), grain size increases into coarser sands, plant material is more abundant, and paleoenvironment is interpreted to have been above fair-weather wave base. The “Muddy Member” consists mostly of mudstone with some interbeds of silt or very fine sand, deposited at or below mean storm-weather wave base.

## Data analysis

Analyses were conducted using R Statistical Software version 4.1.1^54^ and the Calibrate, Plotrix, logistf, and Vegan Packages. Species’ habitat preferences within the Wiscoy Formation were evaluated using non-metric multidimensional scaling (NMDS)^34,35,55^ based on species’ proportional abundances within samples using Bray–Curtis similarity in three dimensions (stress = 0.1245). To separate the effects of onshore-offshore habitat from the effects of opportunistic species blooms for purposes of the regression analysis, the NMDS results were rotated slightly in Fig. 3, which can be justified since the orientation of the point cloud in the original NMDS is based on statistical properties of the data instead of biological properties^34^. The rotation had no effects on the results; the original NMDS is shown in Supplementary Figure S3. The onshore-offshore habitat preference was determined for each species from the NMDS axis 1 score (Fig. 3).

Sensitivity to climate change is often assessed in fossil species using paleolatitudinal distribution; although it does not indicate absolute temperature tolerance, it has been useful as a proxy for relative adaptation to warmer versus cooler temperatures^1,5^. However, the absence of high-latitude faunas in the Frasnian makes it difficult to apply this approach to Frasnian marine taxa for assessing adaptation to cooler waters. In addition, paleogeographic reconstructions are, in some cases, less certain in the Paleozoic than in later times. To address this, we used two different approaches to gauge relative temperature sensitivity. We use both metrics in an attempt to be conservative; that is, if these two independent metrics are both associated with extinction, it should offer a greater degree of support for a true relationship.

For the first metric (species-level), we identified species in the Wiscoy that also occurred in basins from western and central North America by comparison with taxonomic works^41,52^ and museum collections. These other basins are reconstructed at slightly lower latitudes than the Appalachian Basin in the Late Devonian (latitudes were initially taken from the Paleobiology Database, but are consistent with other reconstructions). The potential weakness of this method is that these paleolatitudinal differences are not always large (e.g., Iowa), and it is not known how they translate to temperature. In a few cases, our biogeographic assignments differ slightly from those of other studies^50^ due to differences in taxonomic treatment (e.g., our splitting Wiscoy *Spinatrypa* into two species).

We also inferred relative temperature tolerance at the order level, given previous reports of strong low-latitude preference of some orders^41^. The goal of this metric was to characterize the relative tendency of members of these orders to occur at low versus high latitudes; if an order rarely occurs at high latitudes, it suggests a limited ability to adapt to cooler temperatures. The values obtained are meaningful in a relative sense; that is, they indicate a tendency to occur at high or low latitudes relative to the other orders in the analysis, and only the relative values of the metric are important for subsequent analyses. Each species within the same taxonomic order was given the same value; in reality, species within an order surely varied in thermal tolerance, so this metric is admittedly coarse.

Due to the lack of high-latitude Frasnian collections, we ran the analysis twice. First, we used paleolatitudinal distributions during the Early-Middle Devonian, when higher-latitude collections were available, if not particularly common. This analysis assumes that the latitudinal distributions of the orders did not change substantially between the Early-Middle Devonian and the Frasnian. This assumption appears reasonable; the order-level metrics are strongly correlated between the Early Devonian, Middle Devonian, and Frasnian (despite the lack of high-latitude Frasnian data) (Supplementary Fig. S2), suggesting general stability in relative thermal tolerances. Combining the Early and Middle Devonian increased sample sizes, but it also mixed collections from times with slightly different climates; however, the strong correlation between values from the Early and Middle Devonian (Supplementary Fig. S2) suggests that this will not affect the results. Second, we used the paleolatitudinal distributions during the Frasnian, which are more obviously relevant, but which do not include higher-latitude collections. The main text shows the results based on the Early-Middle Devonian paleolatitude ranges (Fig. 2b, Table 1). The results based on the Frasnian were largely consistent and are shown in Supplementary Table S6 and Supplementary Table S7.

We downloaded brachiopod occurrences entered before 1 October 2021 from the Paleobiology Database^56^, combined occurrences from the northern and southern hemispheres, excluded Siberian collections due to some uncertainty in its reconstruction, and excluded orders not represented in the regression analysis of the Wiscoy fossils (see Fig. 2 for the orders included). Then, we tabulated the number of occurrences of each order within 10° bins, and calculated the proportion of occurrences in each bin that belonged to each order. The paleolatitude index for each order was calculated as a weighted average: if *p*_*ij*_ is the proportion of occurrences in the *j*th bin that belong to the *i*th order, and *m*_*j*_ is the midpoint of the *j*th bin (i.e., 5°, 15°, etc.), then the mean paleolatitude for order *i* is ${\sum }_{j=1}^{9}{p}_{ij}{m}_{j}/{\sum }_{j=1}^{9}{p}_{ij}$. For the regression analysis, these two metrics of temperature tolerance (species- and order level) were combined into a composite metric by converting each to z-scores and summing the two values for each species.

Body size measurements were obtained for at least 20 individuals of each species, or for all available specimens if fewer than 20 were present, and the geometric mean of length and width was calculated. Shell thickness, the third potential body size measurement, was excluded because it could not be obtained from some moldic fossils where shell material was altogether absent. Abundance was calculated as the log of the mean proportional abundance for all specimens of each species. Other measures of abundance (e.g., number of samples in which a species occurs) were highly correlated with this measure.

To test each parameter for importance towards species extinction, an exact logistic regression was run for all possible model combinations of predictor variables including our composite paleolatitude metric, onshore-offshore habitat preference, body size, and log mean proportional abundance (Table 1). Univariate tests comparing victims and survivors of the first extinction pulse for each parameter were conducted with Mann Whitney tests^57^, and a Fisher test^58^ was applied to species occurring at a lower latitude (Supplementary Table S5). Firth’s method was chosen due to the separation within our data and small sample size of species^31^. Analyses were run twice, with *S. hystrix* and *S. amanaensis* treated as victims or as survivors of the first extinction pulse (Table 1).

## Trace metals

Trace metal proxies have been used previously to assess oxygen levels during the extinction pulses in New York^8,9,38^. We extended these studies to several additional localities for the first extinction pulse using XRF and ICP analyses to detect trace metal concentrations. For comparability with previous studies, we present Mo concentrations; concentrations between 2 and 30 ppm were taken to indicate dysoxia, between 30 and 100 ppm indicated intermittent anoxia or euxinia, and greater than 100 ppm indicated permanent anoxia^59^. We also calculated enrichment factors of Mo, U, and V with respect to Ti, following Ref.^9^ and references therein (Supplementary Table S2).

## Supplementary Information

Supplementary Information 1.

Supplementary Information 2.

Supplementary Information 3.

## Data Availability

The fossil data that support this study are available in the Supplementary Material. The authors declare that all other data supporting the findings of this study are available within the paper and its supplementary information files.
