# Supplementary material for: Accelerated mass extinction in an isolated biota during Late Devonian climate changes
Source: Sci Rep. 2021 Dec 21;11:24366. doi: 10.1038/s41598-021-03510-6 (PMC8692332; doi:10.1038/s41598-021-03510-6)
Supplement: Supplementary file 3 — Supplementary Information 3. [file 41598_2021_3510_MOESM3_ESM.docx]

**Supplementary Information**

**Accelerated mass extinction in an isolated biota during Late Devonian climate changes** Jaleigh Q. Pier ^a,b,*^, Sarah K. Brisson^c^, J. Andrew Beard^c^, Michael T. Hren^c^, and Andrew M. Bush^a,c^

**
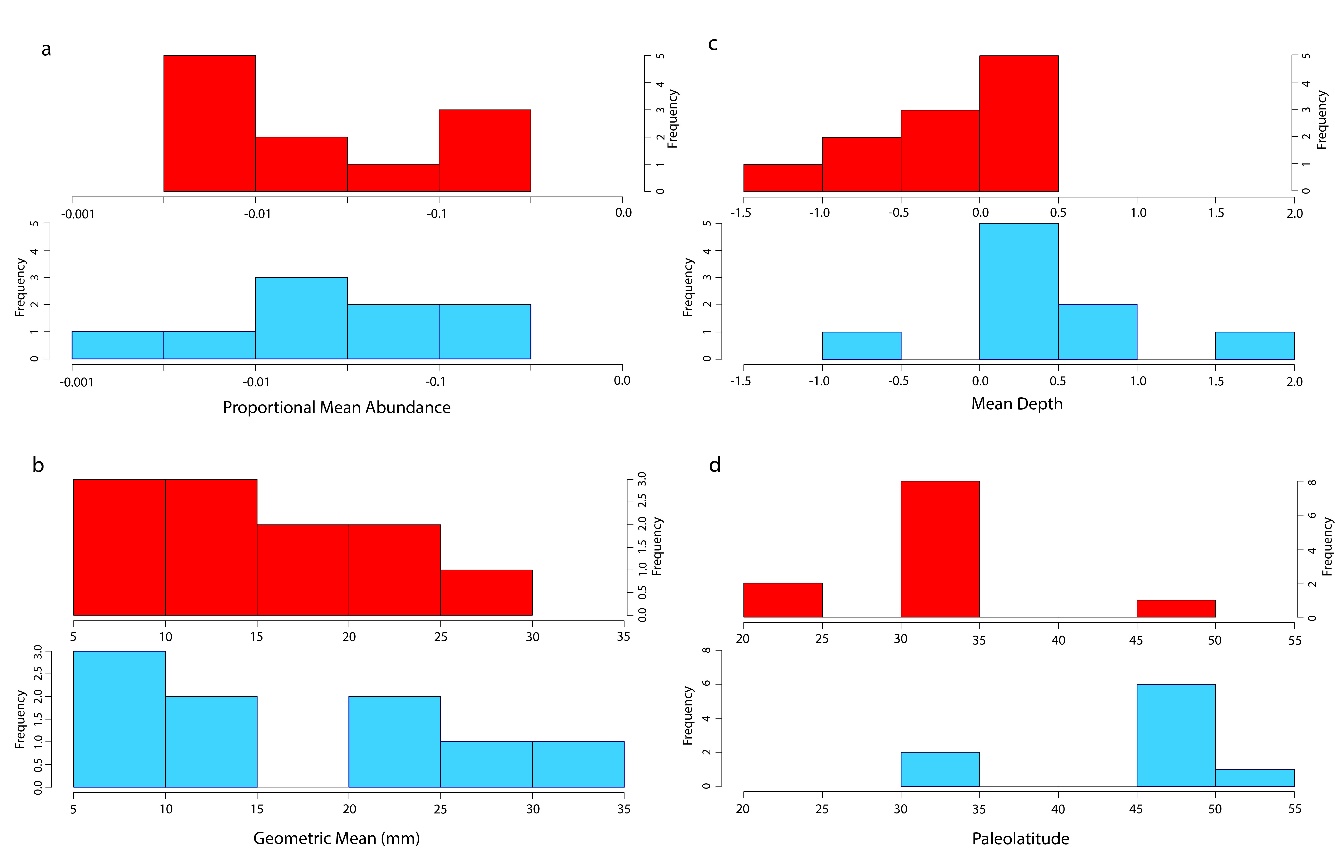
**

Figure S1. Univariate distributions of the four predictor variables for victims (red) and survivors (blue) of the first extinction pulse. (**a**) Mean relative abundance, on a log scale. (**b**) Body size (geometric mean of length and width). (**c**) Mean NMDS 1 value (onshore – offshore habitat preference). (**d**) Order-level paleolatitude index.


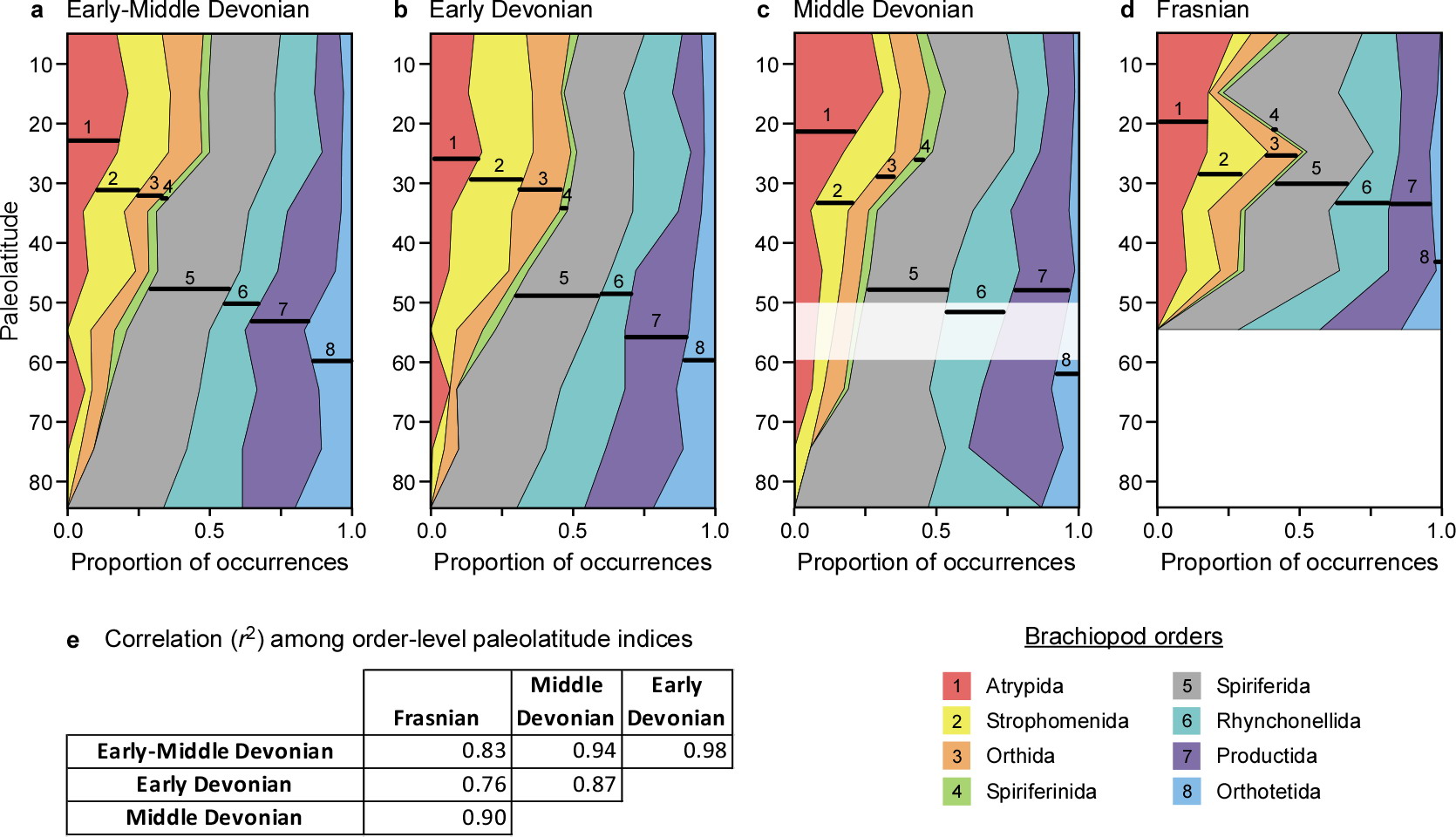


Figure S2. Paleolatitudinal distribution for brachiopod orders during the Devonian (data for northern and southern hemispheres combined). Black lines indicate the weighted averages (see Methods for details of calculation). Data downloaded from the Paleobiology Database^1^. (**a**) Paleolatitudinal distribution for brachiopod orders during the Early-Middle Devonian included in the regression analysis. (**b**) Paleolatitudinal distribution for brachiopod orders during the Early Devonian. (**c**) Paleolatitudinal distribution for brachiopod orders during the Middle Devonian. (**d**) Paleolatitudinal distribution of brachiopod orders during the Frasnian, included in regression results shown in Table S6. White space indicates lack of records. (**e**) Correlations for paleolatitudinal distributions of brachiopod orders panels (**a**)-(**d**).

**
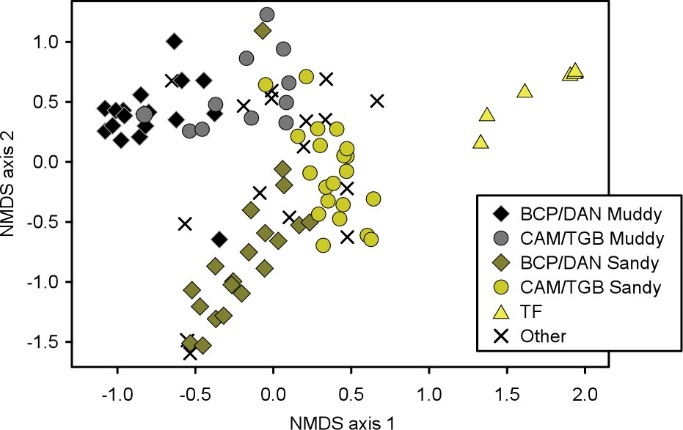
**

Figure S3. Unrotated NMDS results (see Methods).

Table S1. Latitude and longitude of sampling locations. Names are only given for measured sections.

| Locality | Latitude | Longitude | Section Name |
| --- | --- | --- | --- |
| **ADR** | N 42˚15.430' | W 77˚32.156' |  |
| **BCP** | N 42˚21.810' | W 77˚38.675' | Big Creek |
| **BPH** | N 42˚04.394' | W 77˚17.953' |  |
| **CAM** | N 42˚12.008' | W 77˚26.219' | Cameron |
| **CCC** | N 41˚46.643' | W 77˚03.237' |  |
| **CML 1** | N 42˚10.275' | W 77˚21.697' |  |
| **DAN** | N 42˚29.615' | W 77˚39.920' | Dansville |
| **HAN** | N 42˚15.378' | W 77˚29.957' |  |
| **HNS** | N 42˚17.649' | W 77˚38.678' |  |
| **IG** | N 42°40.156' | W 78°45.137’ | Irish Gulf |
| **MFT** | N 41˚48.607' | W 77˚04.369' |  |
| **NCL** | N 41˚59.258' | W 77˚10.326' |  |
| **NYP** | N 42˚00.417' | W 77˚07.800' |  |
| **PUP** | N 41˚57.769' | W 77˚07.254' |  |
| **SCL** | N 41˚58.896' | W 77˚08.951' |  |
| **TF** | N 41˚48.728' | W 76˚30.133' |  |
| **TGB** | N 41˚54.423' | W 77˚09.715' | Tioga, Rt. 287 |
| **WAL** | N 42°32.294′ | W 79°10.133′ | Walnut Creek |
| **WCR** | N 42˚00.670' | W 77˚08.248' |  |
| **WF** | N 42°41.507' | W 78°41.493' | West Falls |
| **WSC** | N 42°30.280′ | W 78°05.014′ | Wiscoy Creek |

Table S2. Trace metal concentrations from the Pipe Creek Formation (first extinction pulse).

| Locality | Source | Method | Sample | Mo (ppm) | U (ppm) | V (ppm) | Ti  (ppm) | Mo (EF) | U  (EF) | V  (EF) |
| --- | --- | --- | --- | --- | --- | --- | --- | --- | --- | --- |
| **TF** | This paper | XRF | TF 23 | 0 | 0 | 49.42 | 2281.06 | 0.00 | 0.00 | 0.77 |
| **TF** | This paper | XRF | TF 22 | 0 | 0 | 125.74 | 6206.95 | 0.00 | 0.00 | 0.72 |
| **TF** | This paper | XRF | TF 21 | 0 | 0 | 133.22 | 5885.32 | 0.00 | 0.00 | 0.80 |
| **TF** | This paper | XRF | TF 20 | 0 | 0 | 132.6 | 6634.81 | 0.00 | 0.00 | 0.71 |
| **TF** | This paper | XRF | TF 19 | 0 | 6.46 | 152.34 | 5882.8 | 0.00 | 1.37 | 0.92 |
| **TF** | This paper | XRF | TF 18 | 0 | 0 | 120.15 | 5526.02 | 0.00 | 0.00 | 0.77 |
| **TF** | This paper | XRF | TF 17 | 0 | 0 | 128.71 | 5386.02 | 0.00 | 0.00 | 0.85 |
| **TF** | This paper | XRF | TF 16 | 0 | 0 | 151.1 | 5792.68 | 0.00 | 0.00 | 0.92 |
| **TF** | This paper | XRF | TF 15 | 0 | 6.94 | 169.15 | 5921.91 | 0.00 | 1.46 | 1.01 |
| **TF** | This paper | XRF | TF 14 | 0 | 0 | 157.04 | 5745.36 | 0.00 | 0.00 | 0.97 |
| **TF** | This paper | XRF | TF 13 | 0 | 0 | 171.25 | 5682.68 | 0.00 | 0.00 | 1.07 |
| **TF** | This paper | XRF | TF 12 | 0 | 0 | 147.96 | 5598.7 | 0.00 | 0.00 | 0.94 |
| **TF** | This paper | XRF | TF 11 | 0 | 0 | 141.78 | 5337.54 | 0.00 | 0.00 | 0.94 |
| **TF** | This paper | XRF | TF 10 | 0 | 0 | 138.66 | 5646.55 | 0.00 | 0.00 | 0.87 |
| **TF** | This paper | XRF | TF 9 | 3.6 | 0 | 138.99 | 5771.02 | 1.10 | 0.00 | 0.85 |
| **TF** | This paper | XRF | TF 8 | 3.6 | 0 | 139.91 | 5992.26 | 1.06 | 0.00 | 0.83 |
| **TF** | This paper | XRF | TF 7 | 3.78 | 0 | 139.14 | 5478.15 | 1.22 | 0.00 | 0.90 |
| **TF** | This paper | XRF | TF 6 | 0 | 0 | 132.89 | 5532.97 | 0.00 | 0.00 | 0.85 |
| **TF** | This paper | XRF | TF 5 | 0 | 5.45 | 107.96 | 5172.65 | 0.00 | 1.31 | 0.74 |
| **TF** | This paper | XRF | TF 4 | 0 | 0 | 117.09 | 4801.79 | 0.00 | 0.00 | 0.86 |
| **TF** | This paper | XRF | TF 3 | 0 | 6.45 | 133.86 | 5510.85 | 0.00 | 1.46 | 0.86 |
| **TF** | This paper | XRF | TF 2 | 0 | 0 | 153.24 | 6082.37 | 0.00 | 0.00 | 0.89 |
| **TF** | This paper | XRF | TF 1 | 0 | 0 | 87.18 | 3734.27 | 0.00 | 0.00 | 0.83 |
| **TGB** | This paper | XRF | A 15 | 0 | 0 | 93.01 | 3562.97 | 0.00 | 0.00 | 0.92 |
| **TGB** | This paper | XRF | A 16 | 0 | 0 | 88.48 | 3317.02 | 0.00 | 0.00 | 0.94 |
| **TGB** | This paper | XRF | A 17 | 0 | 0 | 150.08 | 5532.63 | 0.00 | 0.00 | 0.96 |
| **TGB** | This paper | XRF | A 18 | 0 | 11.59 | 159.53 | 5504.22 | 0.00 | 2.62 | 1.03 |
| **TGB** | This paper | XRF | A 19 | 0 | 9.76 | 168.91 | 5438.72 | 0.00 | 2.23 | 1.10 |
| **TGB** | This paper | XRF | A 20 | 0 | 0 | 110.7 | 4120.17 | 0.00 | 0.00 | 0.95 |
| **TGB** | This paper | XRF | A 21 | 0 | 9.78 | 162.79 | 5458.11 | 0.00 | 2.23 | 1.06 |
| **TGB** | This paper | XRF | TGB 15 | 3.74 | 0 | 164.69 | 5670.35 | 1.17 | 0.00 | 1.03 |
| **TGB** | This paper | XRF | TGB 15 | 2.95 | 0 | 192.9 | 5592.05 | 0.93 | 0.00 | 1.22 |
| **TGB** | This paper | XRF | A 22 | 0 | 9.3 | 189.37 | 5380.44 | 0.00 | 2.15 | 1.25 |
| **TGB** | This paper | XRF | A 23 | 0 | 9.65 | 172.05 | 5333.61 | 0.00 | 2.25 | 1.14 |
| **TGB** | This paper | XRF | A 24 | 0 | 7.92 | 164.42 | 5349.17 | 0.00 | 1.84 | 1.09 |
| **TGB** | This paper | XRF | A 25 | 0 | 11.24 | 181.07 | 5445.89 | 0.00 | 2.57 | 1.18 |
| **TGB** | This paper | XRF | A 26 | 0 | 11.12 | 191.05 | 5278.57 | 0.00 | 2.62 | 1.28 |
| **CAM** | This paper | XRF | CAM 60 | 0 | 0 | 67.52 | 4028.39 | 0.00 | 0.00 | 0.59 |
| **CAM** | This paper | XRF | CAM 61 | 3.14 | 0 | 165.55 | 5606.16 | 0.99 | 0.00 | 1.04 |
| **CAM** | This paper | XRF | CAM 63 | 0 | 0 | 194.38 | 5757.55 | 0.00 | 0.00 | 1.19 |
| **CAM** | This paper | XRF | CAM 64 | 0 | 0 | 164.85 | 5680.05 | 0.00 | 0.00 | 1.03 |
| **CAM** | Boyer et al. 2021 | ICP-MS | 1 | 0.3 | 35 | 3.1 |  |  |  |  |
| **CAM** | Boyer et al. 2021 | ICP-MS | 2 | 2.3 | 66 | 4.0 |  |  |  |  |
| **CAM** | Boyer et al. 2021 | ICP-MS | 3.5 | 0 |  | 2.3 |  |  |  |  |
| **CAM** | Boyer et al. 2021 | ICP-MS | 3.9 | 0 |  | 2.4 |  |  |  |  |
| **CAM** | Boyer et al. 2021 | ICP-MS | 4.0 | 0 |  | 1.7 |  |  |  |  |
| **CAM** | Boyer et al. 2021 | ICP-MS | 4.7 | 2 |  | 1.8 |  |  |  |  |
| **CAM** | Boyer et al. 2021 | ICP-MS | 10 | 0 |  | 1.5 |  |  |  |  |
| **BCP** | This paper | XRF | BCP PC 1 | 7.71 | 0 | 153.63 | 5215.11 | 2.62 | 0.00 | 1.04 |
| **BCP** | This paper | XRF | BCP PC 2 | 0 | 13.35 | 173.29 | 4864.14 | 0.00 | 3.41 | 1.26 |
| **BCP** | This paper | XRF | BCP PC 3 | 0 | 8.54 | 132.51 | 4764.2 | 0.00 | 2.23 | 0.98 |
| **BCP** | This paper | XRF | BCP PC 4 | 0 | 16.66 | 164.18 | 5237.07 | 0.00 | 3.95 | 1.11 |
| **BCP** | This paper | XRF | BCP PC 5 | 0 | 8.21 | 131.12 | 4400.87 | 0.00 | 2.32 | 1.05 |
| **BCP** | This paper | XRF | BCP PC 6 | 0 | 0 | 139.54 | 4798.87 | 0.00 | 0.00 | 1.03 |
| **BCP** | This paper | XRF | BCP PC 7 | 0 | 0 | 181.78 | 3925.24 | 0.00 | 0.00 | 1.64 |
| **BCP** | This paper | XRF | BCP PC 8 | 0 | 9.27 | 189.29 | 5800.71 | 0.00 | 1.99 | 1.15 |
| **BCP** | Boyer et al. 2021 | ICP-MS | 0 | 2.9 | 135 | 4.1 |  |  |  |  |
| **BCP** | Boyer et al. 2021 | ICP-MS | 1 | 1.84 | 73 | 3.2 |  |  |  |  |
| **BCP** | Boyer et al. 2021 | ICP-MS | 2 | 2.32 | 32 | 2.5 |  |  |  |  |
| **BCP** | Boyer et al. 2021 | ICP-MS | 3 | 1.64 | 71 | 3.2 |  |  |  |  |
| **BCP** | Boyer et al. 2021 | ICP-MS | 4 | 2.36 | 98 | 2.9 |  |  |  |  |
| **BCP** | Boyer et al. 2021 | ICP-MS | 5 | 1.18 | 55 | 2.2 |  |  |  |  |
| **BCP** | Boyer et al. 2021 | ICP-MS | 5.8 | 0.75 | 46 | 2.3 |  |  |  |  |
| **WSC** | This paper | XRF | Wisc PC 0-10 | 5.41 | 8.13 | 174 | 5562.87 | 1.72 | 1.82 | 1.11 |
| **WSC** | This paper | XRF | Wisc PC 40 | 0 | 7.16 | 129.46 | 5644.52 | 0.00 | 1.58 | 0.81 |
| **WSC** | This paper | XRF | Wisc PC 70 | 8.78 | 11.83 | 172.39 | 5524.51 | 2.81 | 2.66 | 1.10 |
| **WSC** | This paper | XRF | Wisc PC 100 | 0 | 6.5 | 138.02 | 5719.77 | 0.00 | 1.41 | 0.85 |
| **WSC** | This paper | XRF | Wisc PC 180 | 5.02 | 8.42 | 184.16 | 5078.92 | 1.75 | 2.06 | 1.28 |
| **WSC** | This paper | XRF | Wisc PC 240 | 0 | 10.25 | 173.32 | 5123.92 | 0.00 | 2.49 | 1.20 |
| **WSC** | This paper | XRF | Wisc PC 310 | 5.89 | 11.78 | 171.39 | 5194.52 | 2.01 | 2.82 | 1.17 |
| **IG** | Boyer et al. 2021 | ICP-MS | 20 | 4 | 49 | 2.8 |  |  |  |  |
| **IG** | Boyer et al. 2021 | ICP-MS | 25 | 5 | 94 | 3.0 |  |  |  |  |
| **IG** | Boyer et al. 2021 | ICP-MS | 45 | 5 | 81 | 3.4 |  |  |  |  |
| **IG** | Boyer et al. 2021 | ICP-MS | 50 | 5 | 89 | 3.4 |  |  |  |  |
| **IG** | Boyer et al. 2021 | ICP-MS | 55 | 6 | 74 | 3.5 |  |  |  |  |
| **IG** | Boyer et al. 2021 | ICP-MS | 60 | 5 | 51 | 3.7 |  |  |  |  |
| **IG** | Boyer et al. 2021 | ICP-MS | 65 | 3 | 75 | 3.6 |  |  |  |  |
| **IG** | Boyer et al. 2021 | ICP-MS | 70 | 3 | 61 | 3.4 |  |  |  |  |
| **IG** | Boyer et al. 2021 | ICP-MS | 75 | 3 | 84 | 2.8 |  |  |  |  |
| **IG** | Boyer et al. 2021 | ICP-MS | 85 | 3 | 86 | 2.6 |  |  |  |  |
| **IG** | Boyer et al. 2021 | ICP-MS | 135 | 3 | 80 | 2.5 |  |  |  |  |
| **WF** | Boyer et al. 2021 | ICP-MS | -1 | 4 | 138 | 3.6 |  |  |  |  |
| **WF** | Boyer et al. 2021 | ICP-MS | 0 | 19 | 141 | 3.9 |  |  |  |  |
| **WF** | Boyer et al. 2021 | ICP-MS | 5 | 20 | 139 | 4.8 |  |  |  |  |
| **WF** | Boyer et al. 2021 | ICP-MS | 24 | 9 | 146 | 5.1 |  |  |  |  |
| **WF** | Boyer et al. 2021 | ICP-MS | 28 | 11 | 100 | 5.1 |  |  |  |  |
| **WF** | Boyer et al. 2021 | ICP-MS | 65 | 5 | 148 | 4.2 |  |  |  |  |
| **WF** | Boyer et al. 2021 | ICP-MS | 70 | 5 | 113 | 3.8 |  |  |  |  |
| **WF** | Boyer et al. 2021 | ICP-MS | 75 | 5 | 144 | 3.9 |  |  |  |  |
| **WF** | Boyer et al. 2021 | ICP-MS | 80 | 4 | 146 | 3.5 |  |  |  |  |
| **WF** | Boyer et al. 2021 | ICP-MS | 90 | 4 | 144 | 3.2 |  |  |  |  |
| **WF** | Boyer et al. 2021 | ICP-MS | 95 | 5 | 135 | 3.0 |  |  |  |  |
| **WF** | Boyer et al. 2021 | ICP-MS | 100 | 4 | 131 | 2.7 |  |  |  |  |
| **WF** | Boyer et al. 2021 | ICP-MS | 110 | 5 | 124 | 3.3 |  |  |  |  |
| **WF** | Boyer et al. 2021 | ICP-MS | 115 | 6 | 127 | 3.1 |  |  |  |  |
| **WF** | Boyer et al. 2021 | ICP-MS | 120 | 7 | 131 | 3.2 |  |  |  |  |
| **WF** | Boyer et al. 2021 | ICP-MS | 125 | 5 | 132 | 3.2 |  |  |  |  |
| **WF** | Boyer et al. 2021 | ICP-MS | 130 | 4 | 138 | 3.4 |  |  |  |  |
| **WF** | Boyer et al. 2021 | ICP-MS | 135 | 4 | 151 | 3.4 |  |  |  |  |
| **WF** | Boyer et al. 2021 | ICP-MS | 140 | 5 | 135 | 3.4 |  |  |  |  |
| **WF** | Boyer et al. 2021 | ICP-MS | 150 | 5 | 145 | 3.2 |  |  |  |  |
| **WF** | Boyer et al. 2021 | ICP-MS | 176 | 4 | 141 | 2.8 |  |  |  |  |
| **WF** | Boyer et al. 2021 | ICP-MS | 179 | 4 | 139 | 2.9 |  |  |  |  |
| **WF** | Boyer et al. 2021 | ICP-MS | 200 | 4 | 131 | 3.1 |  |  |  |  |
| **WF** | Boyer et al. 2021 | ICP-MS | 220 | 5 | 123 | 3.2 |  |  |  |  |
| **WF** | Boyer et al. 2021 | ICP-MS | 235 | 5 | 75 | 3.2 |  |  |  |  |
| **WF** | Boyer et al. 2021 | ICP-MS | 240 | 5 | 128 | 3.1 |  |  |  |  |
| **WF** | Boyer et al. 2021 | ICP-MS | 260 | 4 | 103 | 3.4 |  |  |  |  |
| **WF** | Boyer et al. 2021 | ICP-MS | 280 | 5 | 100 | 3.5 |  |  |  |  |
| **WAL** | Lash 2017 | XRF | LKW | 4 |  |  |  |  |  |  |
| **WAL** | Lash 2017 | XRF | LKW | 15 |  |  |  |  |  |  |
| **WAL** | Lash 2017 | XRF | LKW | 26 |  |  |  |  |  |  |
| **WAL** | Lash 2017 | XRF | LKW | 27 |  |  |  |  |  |  |
| **WAL** | Lash 2017 | XRF | LKW | 33 |  |  |  |  |  |  |
| **WAL** | Lash 2017 | XRF | LKW | 19 |  |  |  |  |  |  |
| **WAL** | Lash 2017 | XRF | LKW | 13 |  |  |  |  |  |  |
| **WAL** | Lash 2017 | XRF | LKW | 12 |  |  |  |  |  |  |
| **WAL** | Lash 2017 | XRF | LKW | 13 |  |  |  |  |  |  |
| **WAL** | Lash 2017 | XRF | LKW | 15 |  |  |  |  |  |  |
| **WAL** | Lash 2017 | XRF | LKW | 17 |  |  |  |  |  |  |
| **WAL** | Lash 2017 | XRF | LKW | 13 |  |  |  |  |  |  |
| **WAL** | Lash 2017 | XRF | LKW | 15 |  |  |  |  |  |  |
| **WAL** | Lash 2017 | XRF | LKW | 14 |  |  |  |  |  |  |
| **WAL** | Lash 2017 | XRF | LKW | 13 |  |  |  |  |  |  |
| **WAL** | Lash 2017 | XRF | LKW | 12 |  |  |  |  |  |  |
| **WAL** | Lash 2017 | XRF | LKW | 11 |  |  |  |  |  |  |
| **WAL** | Lash 2017 | XRF | LKW | 10 |  |  |  |  |  |  |
| **WAL** | Lash 2017 | XRF | LKW | 9 |  |  |  |  |  |  |
| **WAL** | Lash 2017 | XRF | LKW | 5 |  |  |  |  |  |  |
| **WAL** | Lash 2017 | XRF | LKW | 4 |  |  |  |  |  |  |
| **WAL** | Lash 2017 | XRF | LKW | 3 |  |  |  |  |  |  |
| **WAL** | Kelly et al. (2019) | ICP-MS | WCLK 66 | 86.2 | 8 |  |  |  |  |  |
| **WAL** | Kelly et al. (2019) | ICP-MS | WCLK 58 | 54.9 | 7.8 |  |  |  |  |  |
| **WAL** | Kelly et al. (2019) | ICP-MS | WCLK 53 | 61.7 | 8.5 |  |  |  |  |  |
| **WAL** | Kelly et al. (2019) | ICP-MS | WCLK 33 | 53.5 | 10 |  |  |  |  |  |

Table S3. Values for each species incorporated into the regression analysis. Several species were excluded from the regression analysis due to small sample size or poor preservation (see Methods). Extinct: 0 = no, 1 = yes. Paleolatitude Index (Species-Level): 0 = extends to lower-latitude basins, 1 = does not extend to lower-latitude basins. Paleolatitude Index (Composite Metric): sum of z-scores for “Paleolatitude Index (Species-Level)” and “Paleolatitude Index (Order-Level)” for each species. Proportion of Total Specimens: proportion of each species out of all fossil specimens included. Per Sample Mean Proportion: average proportion of each species per sample. Log_10_ (Per Sample Mean Proportion): Log of Per Sample Mean Proportion. Paleolatitude index (Order-Level): average paleolatitude for each brachiopod order based on Paleobiology Database data from the Early-Middle Devonian. Onshore-offshore habitat preference: NMDS score on Axis 1 for each species. Body Size: geometric mean of length and width for at least 20 individuals for each species. Species abbreviations listed in Table S4.

| Species | Extinct | Paleolatitude Index (Species-Level) | Paleo-latitude Index (Composite Metric) | Proportion of Total Specimens | Per Sample Mean Proportion | Log_10_(Per Sample Mean Proportion) | Paleo-latitude Index (Order-Level) | Onshore-offshore habitat preference | Body Size |
| --- | --- | --- | --- | --- | --- | --- | --- | --- | --- |
| **S.infera** | 1 | 0 | -2.156 | 0.163 | 0.158 | -0.801 | 32.1 | -1.212 | 8.48 |
| **S.impressa** | 0 | 1 | -0.029 | 0.017 | 0.015 | -1.835 | 32.1 | -0.905 | 20.72 |
| **S.amanaensis** | 1 | 0 | -2.156 | 0.038 | 0.047 | -1.332 | 32.1 | -0.656 | 16.20 |
| **S.trulla** | 1 | 0 | -2.922 | 0.007 | 0.006 | -2.233 | 23.76 | -0.576 | 23.90 |
| **D.arcuata** | 1 | 0 | -2.255 | 0.108 | 0.130 | -0.887 | 31.03 | -0.439 | 10.76 |
| **Schizophoria.sp.A** | 1 | 0 | -2.156 | 0.006 | 0.009 | -2.058 | 32.1 | -0.439 | 7.04 |
| **D.cayuta** | 1 | 1 | -0.128 | 0.018 | 0.024 | -1.623 | 31.03 | -0.405 | 13.96 |
| **W.hirsuta** | 0 | 1 | 1.923 | 0.067 | 0.101 | -0.995 | 53.36 | 0.117 | 13.12 |
| **A.gregaria** | 0 | 1 | 1.442 | 0.306 | 0.152 | -0.818 | 48.12 | 0.135 | 5.94 |
| **C.contractum** | 0 | 1 | 1.650 | 0.005 | 0.007 | -2.129 | 50.39 | 0.178 | 9.53 |
| **T.mesacostalis** | 0 | 1 | 1.442 | 0.001 | 0.002 | -2.824 | 48.12 | 0.183 | 11.65 |
| **N.nervosa** | 1 | 1 | -0.128 | 0.002 | 0.005 | -2.294 | 31.03 | 0.238 | 26.37 |
| **R.orestes** | 1 | 0 | -0.685 | 0.004 | 0.009 | -2.035 | 48.12 | 0.302 | 12.09 |
| **C.hamiltonensis** | 1 | 1 | 0.046 | 0.004 | 0.006 | -2.223 | 32.92 | 0.343 | 7.85 |
| **S.hystrix** | 1 | 1 | -0.795 | 0.083 | 0.127 | -0.896 | 23.76 | 0.345 | 17.94 |
| **C.chemungensis** | 0 | 1 | 1.442 | 0.011 | 0.015 | -1.811 | 32.1 | 0.478 | 32.86 |
| **S.coelata** | 1 | 1 | -0.128 | 0.014 | 0.021 | -1.675 | 32.1 | 0.480 | 22.55 |
| **C.inermis** | 0 | 1 | 1.442 | 0.030 | 0.047 | -1.329 | 32.1 | 0.575 | 28.94 |
| **F.chemungensis** | 0 | 1 | 2.505 | 0.014 | 0.021 | -1.662 | 23.76 | 0.701 | 22.54 |
| **C.eximium** | 0 | 1 | 1.650 | 0.078 | 0.061 | -1.217 | 50.39 | 1.871847 | 7.46 |

Table S4. Brachiopod species and abbreviations. See Figure 2A for presence of each species across the two pulses.

| Abbreviation | Species | Order |
| --- | --- | --- |
| *D .arcuata* | *Douvillina arcuata* | Strophomenida |
| *D. cayuta* | *Douvillina cayuta* | Strophomenida |
| *S. coelata* | *Strophonelloides coelata* | Strophomenida |
| *N. nervosa* | *Nervostrophia nervosa* | Strophomenida |
| Chonetid | Chonetid species | Productida |
| *D. walcotti* | *Devonoproductus walcotti* | Productida |
| *W. hirsuta* | *Whidbornella hirsuta* | Productida |
| *F. chemungensis* | *Floweria chemungensis* | Orthotedida |
| *S. infera* | *Stainbrookia infera* | Orthida |
| *S. impressa* | *Schizophoria impressa* | Orthida |
| *S. amanaensis* | *Schizophoria amanaensis* | Orthida |
| *S*. sp. A | *Schizophoria* species A | Orthida |
| *C. contractum* | *Cupularostrum contractum* | Rhynchonellida |
| *K. mesacostalis* | *Katabuporhynchus mesacostalis* | Rhynchonellida |
| *C. eximium* | *Cupularostrum eximium* | Rhynchonellida |
| *S. hystrix* | *Spinatrypa hystrix* | Atrypida |
| *S. trulla* | *Spinatrypa trulla* | Atrypida |
| *P. devoniana* | *Pseudoatrypa devoniana* | Atrypida |
| *A. angelica* | *“Athyris” angelica* | Athyridida |
| *C. inermis* | *Cyrtospirifer inermis* | Spiriferida |
| *C. chemungensis* | *Cyrtospirifer chemungensis* | Spiriferida |
| *R. orestes* | *Rigauxia orestes* | Spiriferida |
| *T. mesacostalis* | *Tylothyris mesacostalis* | Spiriferida |
| *A. gregaria* | *Ambocoelia gregaria* | Spiriferida |
| *C. hamiltonensis* | *Cyrtina hamiltonensis* | Spiriferinidida |
| *P. speciosa* | *Praewaagenoconcha speciosa* | Productida |
| *P. stigmata* | *“Productella” stigmata* | Productida |
| *P. lachrymosa* | *Praewaagenoconcha speciosa* | Productida |
| *S. onustus* | *Semiproductus onustus* | Productida |
| *P. rectispina* | *“Productella” rectispina* | Productida |
| *D. allegania* | *“Dalmanella” allegania* | Orthida |
| *T. leonensis* | *“Thiemella” leonensis* | Orthida |
| *S. planosulcata* | *Spinatrypa planosulcata* | Atrypida |
| *E. multicostata* | *Eumetabolatoechia multicostata* | Rhynchonellida |
| *J. duplicatus* | *Jacoburbirostrum duplicatus* | Rhynchonellida |

Table S5. Summary of univariate tests comparing victims and survivors of the first extinction pulse for each parameter separately. Mann Whitney tests ^2^ were applied to all parameters, except for ‘Paleolatitude Index (Species-Level)’ for which the Fisher Test^3^ was applied. Asterisk marks significant results (*p* < 0.05).

| Parameter | Paleolatitude index (Order-Level) | Body Size | Onshore-offshore habitat preference | % Abundance | Paleolatitude Index (Species-Level) |
| --- | --- | --- | --- | --- | --- |
| **W** | 5.5 | 48 | 31 | 46 |  |
| **p-value** | 0.0008* | 0.941 | 0.175 | 0.824 | 0.0009* |

Table S6. Exact logistic regression results of variables predicting the likelihood of extinction of brachiopod species, with order-level paleolatitude indices based on the Frasnian (see Methods). A. Analyses run with *Spinatrypa hystrix* and *Schizophoria amanaensis* as victims. Odds ratio = 11.46, p-value = 0.022. B. Analyses run with *Spinatrypa hystrix* and *Schizophoria amanaensis* as survivors. Odds ratio = 6.200, p-value = 0.185.

| 1. **Parameter** | **Paleolatitude Index**  **(Composite Metric)** | **Body Size** | **Onshore-offshore Habitat Preference** | **% Abundance** |
| --- | --- | --- | --- | --- |
| Odds Ratio | 0.21 | 1.008 | 1.79 | 0.69 |
| p-value | 0.0034 | 0.9068 | 0.5706 | 0.7206 |
| Confidence Interval | (-5.91, -0.41) | (-0.13, 0.14) | (-1.72, 2.86) | (-4.94, 4.50) |
|  |  |  |  |  |
| 1. **Parameter** | **Paleolatitude Index**  **(Composite Metric)** | **Body Size** | **Onshore-offshore Habitat Preference** | **% Abundance** |
| Odds Ratio | 0.48 | 0.98 | 1.10 | 0.44 |
| p-value | 0.0744 | 0.7522 | 0.9111 | 0.3109 |
| Confidence Interval | (-1.86, 0.06) | (-0.15, 0.11) | (-1.91, 1.82) | (-2.61, 0.76) |

Table S7. Summary of univariate tests comparing victims and survivors of the first extinction pulse for each parameter separately, with order-level paleolatitude indices based on the Frasnian (see Methods). Mann Whitney tests^2^ were applied to all parameters, except for ‘Paleolatitude Index (Species-Level)’ for which the Fisher Test ^3^ was applied. Asterisk marks significant results (*p* < 0.05).

| Parameter | Paleolatitude Index (Order-Level | Body Size | Onshore-offshore habitat preference | % Abundance | Paleolatitude Index (Species-Level) |
| --- | --- | --- | --- | --- | --- |
| **W** | 8.5 | 48 | 31 | 46 |  |
| **p-value** | 0.0018* | 0.941 | 0.175 | 0.824 | 0.0009* |
